# Prophylactic and Therapeutic Efficacy of Human Monoclonal Antibodies against H5N1 Influenza

**DOI:** 10.1371/journal.pmed.0040178

**Published:** 2007-05-29

**Authors:** Cameron P Simmons, Nadia L Bernasconi, Amorsolo L Suguitan, Kimberly Mills, Jerrold M Ward, Nguyen Van Vinh Chau, Tran Tinh Hien, Federica Sallusto, Do Quang Ha, Jeremy Farrar, Menno D de Jong, Antonio Lanzavecchia, Kanta Subbarao

**Affiliations:** 1 Oxford University Clinical Research Unit, Hospital for Tropical Diseases, Ho Chi Minh City, Viet Nam; 2 Institute for Research in Biomedicine, Bellinzona, Switzerland; 3 Laboratory of Infectious Diseases, National Institute of Allergy and Infectious Diseases, National Institutes of Health, Bethesda, Maryland, United States of America; 4 Comparative Medicine Branch, National Institute of Allergy and Infectious Diseases, National Institutes of Health, Bethesda, Maryland, United States of America; 5 Hospital for Tropical Diseases, Ho Chi Minh City, Viet Nam; Mount Sinai School of Medicine, United States of America

## Abstract

**Background:**

New prophylactic and therapeutic strategies to combat human infections with highly pathogenic avian influenza (HPAI) H5N1 viruses are needed. We generated neutralizing anti-H5N1 human monoclonal antibodies (mAbs) and tested their efficacy for prophylaxis and therapy in a murine model of infection.

**Methods and Findings:**

Using Epstein-Barr virus we immortalized memory B cells from Vietnamese adults who had recovered from infections with HPAI H5N1 viruses. Supernatants from B cell lines were screened in a virus neutralization assay. B cell lines secreting neutralizing antibodies were cloned and the mAbs purified. The cross-reactivity of these antibodies for different strains of H5N1 was tested in vitro by neutralization assays, and their prophylactic and therapeutic efficacy in vivo was tested in mice. In vitro, mAbs FLA3.14 and FLD20.19 neutralized both Clade I and Clade II H5N1 viruses, whilst FLA5.10 and FLD21.140 neutralized Clade I viruses only. In vivo, FLA3.14 and FLA5.10 conferred protection from lethality in mice challenged with A/Vietnam/1203/04 (H5N1) in a dose-dependent manner. mAb prophylaxis provided a statistically significant reduction in pulmonary virus titer, reduced associated inflammation in the lungs, and restricted extrapulmonary dissemination of the virus. Therapeutic doses of FLA3.14, FLA5.10, FLD20.19, and FLD21.140 provided robust protection from lethality at least up to 72 h postinfection with A/Vietnam/1203/04 (H5N1). mAbs FLA3.14, FLD21.140 and FLD20.19, but not FLA5.10, were also therapeutically active in vivo against the Clade II virus A/Indonesia/5/2005 (H5N1).

**Conclusions:**

These studies provide proof of concept that fully human mAbs with neutralizing activity can be rapidly generated from the peripheral blood of convalescent patients and that these mAbs are effective for the prevention and treatment of H5N1 infection in a mouse model. A panel of neutralizing, cross-reactive mAbs might be useful for prophylaxis or adjunctive treatment of human cases of H5N1 influenza.

## Abstract 2

Cameron Simmons and colleagues provide proof of concept that human monoclonal antibodies with neutralizing activity can be rapidly generated from peripheral blood of convalescent patients and are effective in preventing and treating H5N1 infection in a mouse model.

## Editors' Summary

**Background.:**

Every year, millions of people catch influenza, a viral disease of the nose, throat, and airways. Although most recover, influenza outbreaks (epidemics) kill about half a million people annually. Epidemics occur because small but frequent changes in the viral proteins (antigens) to which the immune system responds mean that an immune response produced one year provides only partial protection against influenza the next year. Human flu viruses also occasionally appear that contain major antigenic changes. People have little or no immunity to such viruses (which often originate in animals or birds), so these viruses can start deadly pandemics—global epidemics. The Spanish flu pandemic in 1918/9, Asian flu in 1957, and Hong Kong flu in 1968 all killed millions. Experts believe that another pandemic is overdue and may be triggered by the avian H5N1 influenza virus—the name indicates that this bird virus carries type 5 hemagglutinin and type 1 neuraminidase, the two major flu antigens. H5N1, which rapidly kills infected birds, is now present in flocks around the world and, since 1997, it has caused 258 cases of human flu and 153 deaths. People have caught H5N1 through close contact with infected birds but, luckily, H5N1 rarely passes between people.

**Why Was This Study Done?:**

H5N1 might acquire the ability to move between people and start a human influenza pandemic at any time. Some of the H5N1 viruses are resistant to the antiviral drugs used to treat flu and there will inevitably be a lag of some months between the emergence of a human pandemic H5N1 strain and the bulk production of a vaccine effective against it. Thus, new preventative and therapeutic strategies are needed to combat human infections with H5N1. One possibility is passive immunotherapy—treating people with antibodies (proteins that recognize antigens) that can stop H5N1 from infecting cells (so-called neutralizing antibodies). In this study, the researchers have generated neutralizing human monoclonal antibodies (laboratory-produced preparations that contain one type of human antibody) and tested their ability to halt viral growth in mice infected with H5N1.

**What Did the Researchers Do and Find?:**

Patients who have survived infection with H5N1 make neutralizing antibodies, so the researchers isolated and immortalized the immune cells making these antibodies from the patients' blood. They grew up each cell separately and purified the antibody that the cells made. These monoclonal antibodies were then tested for their ability to neutralize H5N1 and other flu viruses in the laboratory. The researchers identified several that neutralized the H5N1 strain with which the patients were originally infected and chose two for further study. In the test tube, the four antibodies neutralized closely related H5N1 viruses and an H5N1 virus from a different lineage (clade) that has also caused human disease, in addition to the original H5N1 virus, although with different efficacies. In mice, the antibodies provided protection from infection with the original virus when given a day before or one to three days after infection. Three antibodies also partly protected the mice against H5N1 from a different clade. Finally, the researchers showed that the antibodies protected mice by limiting viral replication, by lessening the deleterious effects of the virus in the lungs, and by stopping viral spread out of the lungs.

**What Do These Findings Mean?:**

These results indicate that passive immunotherapy with human monoclonal antibodies could help to combat avian H5N1 if (or when) it starts a human pandemic. Passive immunotherapy is already used to prevent infections with several other viruses. In addition, a crude form of the approach—early treatment of patients with plasma (the liquid portion of blood) from convalescent patients—halved the death rate during the Spanish flu pandemic. Large amounts of pure monoclonal antibodies can be relatively easily made for clinical use, and this study indicates that some monoclonal antibodies neutralize H5N1 viruses from different clades. The researchers sound a note of caution, however: Before passive immunotherapy can help to halt an H5N1 pandemic, they warn, the monoclonal antibodies will have to be tested to see whether they can neutralize not only all the currently circulating H5N1 viruses but also any emerging pandemic versions, which might be antigenically distinct.

**Additional Information.:**

Please access these Web sites via the online version of this summary at http://dx.doi.org/10.1371/journal.pmed.0040178.

- US Centers for Disease Control and Prevention information about influenza for patients and professionals including key facts about avian influenza
- US National Institute of Allergy and Infectious Disease feature on seasonal, avian, and pandemic flu
- World Health Organization factsheet on influenza and information on avian influenza, including latest figures for confirmed human cases
- UK Health Protection Agency information on seasonal, avian, and pandemic influenza
- Wikipedia pages on passive immunity and monoclonal antibodies (note: Wikipedia is an online encyclopedia that anyone can edit)

## Introduction

The continued circulation of highly pathogenic avian influenza (HPAI) strains of subtype H5N1, and occasional coincident cases of human infection (274 patients as of 19 February 2007, with 167 fatalities), has triggered international public health concern. On the basis of haemagglutinin (HA) sequences, these circulating HPAI H5N1 viruses fall into different lineages, termed clades; viruses isolated in Viet Nam and Indonesia in 2004 and 2005, respectively, were designated as reference strains for Clades I and II [1]. The HA sequences of Clade I and Clade II viruses differ by 4% to 5% at the amino acid level and the viruses of the two clades are antigenically distinguishable. The H5N1 viruses are not efficiently transmitted from person to person. Potentially, a virus capable of efficient human-to-human transmission could result from either adaptation of the HPAI H5N1 viruses and/or reassortment of the H5N1 virus genome with that of a circulating human influenza virus. Widespread dissemination of such a virus could cause significant morbidity and mortality, since humans are generally immunologically naïve to H5 influenza subtypes.

In humans, overall mortality in HPAI H5N1 infection exceeds 60%, with variation according to the patient's age and the year of infection [2]. The basis for the apparent virulence of HPAI H5N1 strains in humans is relatively poorly understood. In Vietnamese patients, the disease was characterized by severe pneumonia, lymphopenia, high viral loads in the respiratory tract, and hypercytokinemia [3]. Beyond supportive care, treatment options for human patients with H5N1 avian influenza remain limited and are empiric; some H5N1 viruses are resistant to older antiviral agents such as amantadine and rimantadine [4,5], and the clinical efficacy of neuraminidase inhibitors such as oseltamavir and zanamavir has not yet been confirmed in prospective studies. In addition, H5N1 viruses resistant to oseltamavir have been reported [6,7]. Patients who recover from infection possess antibodies that neutralize their infecting virus in vitro, suggesting that antibody-mediated immunity may contribute to resolution of infection [3].

Antibody-based therapy for human patients with H5N1 is a hitherto unexplored, but potentially viable, treatment option. Clinically, antibody therapy using polyclonal and monoclonal antibodies (mAbs) is effectively used as prophylaxis against varicella, hepatitis A, hepatitis B, rabies, and respiratory syncytial virus infections [8]. In the context of influenza, specific mAbs can confer prophylactic and therapeutic protection in mice [9–11]. Passive immunization by vertical acquisition of specific antibodies is also associated with influenza immunity in animal models and in early infancy in humans [12–15]. Of more immediate relevance, transfusion of human blood products from patients recovering from the 1918 “Spanish ‘flu” was associated with a 50% reduction in influenza mortality during the pandemic [16]. Humanized mouse mAbs and equine F(ab′)2 fragments specific for H5N1 have also been used for efficacious prophylaxis and therapy in the mouse model [17,18]. Collectively, these observations suggest that passive antibody therapy against HPAI H5N1 viruses could be a potentially viable, adjunctive treatment option in human cases of H5N1 influenza.

One approach to generating virus-specific neutralizing mAbs is to use a highly efficient method of Epstein-Barr (EBV)-mediated immortalization of memory B cells from convalescent individuals [19]. This approach is rapid and yields stable B cell clones that secrete fully human antibodies that have been selected in the course of an immune response to the pathogen [19]. The aim of this study was to generate human mAbs with neutralizing activity against H5N1 viruses. mAbs were derived from immortalized memory B cells collected from donors who had recovered from H5N1 infection. Four mAbs, with neutralizing activity in vitro, had prophylactic and therapeutic efficacy in mice challenged with HPAI H5N1. These mAbs, and others like them, could have a role in adjunctive treatment of human cases of H5N1 influenza.

## Materials and Methods

### H5N1 Influenza Cases

The four adult blood donors in this study (CL26, CL36, CL114, and CL115) were diagnosed with HPAI H5N1 infection between January 2004 and February 2005 at the Hospital for Tropical Diseases in Ho Chi Minh City, Viet Nam. Diagnosis of HPAI H5N1 influenza infection was made by RT-PCR on respiratory specimens (from all four donors) and culture of H5N1 influenza from respiratory specimens (from donors CL26, CL36, and CL115) [20]. The clinical features of acute disease in three of the four patients (donors CL26, CL36, and CL115) have been described previously (patients #5, #7, and #8 in the online supplement to [7]). During early convalescence (1–4 mo post-illness onset), all patients had detectable neutralizing antibody titers to their autologous virus (median 96, range 32–200). The Scientific and Ethical Committee of the Hospital for Tropical Diseases and the Oxford University Tropical Research Ethical Committee approved the study protocol. All patients provided written informed consent.

### Influenza Viruses

GenBank accession numbers for the genomic sequences of the H5N1 viruses isolated from subjects CL26 (A/Vietnam/CL26/2004), CL36 (A/Vietnam/CL36/2004) and CL115 (A/Vietnam/CL115/2005), have been published previously [3]. Strain A/chicken/Vietnam/VL1/2006 (H5N1) was isolated from a cloacal swab of a chicken in southern Viet Nam in early 2006. The HPAI H5N1 reference viruses A/Vietnam/1203/2004, A/Hong Kong/213/2003, A/Hong Kong/491/1997, A/Vietnam/JPHN/30321/2005, and A/Indonesia/5/2005 were kindly provided by Dr. Nancy Cox, Influenza Division, Centers for Disease Control and Prevention, Atlanta, Georgia, United States. Influenza A/California/7/2004 (H3N2) was kindly provided by Dr. Roland Levandowski, CBER, Food and Drug Administration, Bethesda, Maryland, United States.

### Microneutralization Assays

Screening of supernatants from B cell lines and clones was performed by microneutralization assay using MDCK cells and 100 TCID_50_ (50% tissue culture infectious doses) of A/Vietnam/1203/04 essentially as described previously [21]. Briefly, neat supernatants were incubated with 100 TCID_50_ of virus for 1 h at room temperature prior to addition to monolayers of MDCK cells. Cell monolayers were incubated for a further 3–4 d and examined for cytopathic effect. Determination of endpoint neutralizing antibody titers was performed in a similar fashion, except that plasma or supernatant samples were serially two-fold diluted prior to mixing with 100 TCID_50_ of virus. Plasma samples were tested at a starting dilution of 1:10, while supernatants were tested at a starting dilution of 1:8 and residual infectivity was tested in four wells per dilution. The neutralizing titer was defined as the reciprocal of the highest dilution of serum at which the infectivity of 100 TCID_50_ of the appropriate wild-type (wt) H5N1 virus for MDCK cells was completely neutralized in 50% of the wells. Infectivity was identified by the presence of cytopathic effect on d 4 and the titer was calculated by the Reed-Muench method.

### Immortalization of Memory B Cells and Selection of Neutralizing Clones

Frozen peripheral blood mononuclear cells (PBMCs) were thawed and stained with directly labeled antibodies to CD22 (Pharmingen, http://www.bdbiosciences.com/home) and to immunoglobulin (Ig) M, IgD, and IgA (Jackson ImmunoResearch, http://www.jacksonimmuno.com). CD22+ IgM−, IgD−, IgA− B cells were isolated using a FACSAria (Becton Dickinson, http://www.bd.com) and immortalized at 30 B cells/well in replicate cultures using EBV in the presence of CpG oligodeoxynucleotide 2006 (Mycrosynth, http://www.microsynth.ch) and irradiated allogeneic PBMC, as previously described [19]. Cells were cultured in complete RPMI 1640 supplemented with 10% fetal calf serum (HyClone Laboratories, http://www.hyclone.com). Culture supernatants were harvested after 14 days and assayed for neutralizing activity against 100 TCID_50_ of influenza A/Vietnam/1203/04 (H5N1). Cultures with measurable neutralizing activity were cloned at 0.5 cell/well in the presence of CpG 2006 and irradiated PBMCs. B cell clones were cultured at a high cell density in complete RPMI 1640 10% Ig-depleted fetal calf serum to produce enriched supernatants containing 1–3 mg mAbs/ml. MAbs were also purified on protein G columns (GE Healthcare Europe http://www.gehealthcare.com). The isotype, subclass, and light chain of the mAbs were characterized by ELISA using specific antibodies and HRP-labeled anti-human Ig antibody (Southern Biotechnology, http://www.southernbiotech.com). Antibodies were quantified with reference to a standard certified preparation (Sigma-Aldrich, http://www.sigmaaldrich.com).

### MAbs for Prophylaxis and Therapy in Mice

Groups of 4–8 female BALB/c mice (4–6 wk old, mean weight 18 g) were used in all experiments. Inoculation of mice and tissue harvests were performed in a biosafety cabinet by personnel wearing powered air purifying respirators. Influenza-infected animals were housed in a USDA and CDC accredited biosafety level 3 (BSL3) animal facility in accordance with protocols approved by the NIH Animal Care and Use Committee. To measure prophylactic efficacy, mice were intraperitoneally (i.p.) injected with 1 ml of various antibody preparations or hyperimmune sheep antisera raised against baculovirus expressed HA of A/VN/1203/2004 (H5N1) that was kindly provided by Dr. G. Kemble, Medimmune Vaccines (http://www.medimmune.com). The H5N1 mAbs FLA3.14, FLA5.10, FLD20.19 and FLD21.140 were administered either as purified IgG or as enriched culture supernatant. Control human antibodies were IgG1 mAbs D2.2 or A146, specific for diphtheria toxin and anthrax protective antigen, respectively, and were prepared in the same fashion as the influenza-reactive antibodies. Twenty-four hours after i.p. administration, the mice were bled to collect samples for measurement of neutralizing human mAb titers, then challenged intranasally (i.n.) with 10^5^ TCID_50_ of A/Vietnam/1203/04 (H5N1) or A/Indonesia/5/2005 (H5N1) in 50 μl. Mice were observed and weighed daily before and after viral infection. To determine viral titers following challenge, mice were killed and the lungs, brains, and spleens were aseptically removed. Tissues were homogenized in Leibovitz L-15 medium (Invitrogen, http://www.invitrogen.com) supplemented with antibiotic-antimycotic solution (Gibco, http://www.invitrogen.com) to achieve suspensions of lung (10% w/v), spleen (5% w/v), and brain (10% w/v), which were then titrated on monolayers of MDCK cells in quadruplicate. The viral titer was calculated by the Reed and Muench method and expressed as log_10_ TCID_50_ per gram of tissue.

For therapy against A/Vietnam/1203/04 (H5N1), the mice were first infected i.n. with 5 LD_50_ of A/Vietnam/1203/04, then 24, 48, or 72 h later they were injected i.p. with 1 ml of a mAb preparation. For therapy against A/Indonesia/5/2005 (H5N1), mice were first infected i.n. with 5 LD_50_ of A/Indonesia/5/2005 (H5N1), then 24 h later injected i.p. with 1 ml of a mAb preparation.

### Pathology

Mice were necropsied and the lungs were inflated with 10% neutral buffered formalin and embedded in paraffin, and sections were prepared. Slides were stained with hematoxylin and eosin. For immunohistochemical demonstration of H5 antigen, paraffin sections were prepared and ABC immunohistochemistry was performed using a goat antibody to avian influenza H5 Goat Alpha H5 (NIAID Reference Reagents, BEI Resources, http://www.beiresources.org) diluted at 1:1,000, with a Vector Rabbit Anti-Goat secondary antibody, the Vector ABC Elite label (Vector Laboratories, http://www.vectorlabs.com) diaminobenzidine as the chromogen, and hematoxylin as the counterstain. Lung pathology was evaluated in a semiquantitative manner by a pathologist (JW) blind to the treatment.

### Statistics

Kaplan-Meier survival curves and log rank tests were used to measure differences between treatment arms in prophylactically and therapeutically treated mice. The Mann-Whitney U test was used to measure differences in viral loads in mouse tissues. For statistical purposes, samples with undetectable viral burdens were given the value 1.5 log_10_ TCID/g. All analyses were performed in Stata 8.2 software (StataCorp, http://www.stata.com).

## Results

Blood samples from four Vietnamese adults (CL26, CL36, CL114, and CL115) who had recovered from HPAI H5N1 infection were collected 3–15 mo postinfection. IgG^+^ memory B cells recovered from frozen PBMC were immortalized with EBV. Cultures secreting neutralizing antibodies were identified by a microneutralization assay against the prototype Clade I virus, A/Vietnam/1203/04 (H5N1), and cloned by limiting dilution. Supernatants from approximately 11,000 wells were screened to identify 15 independent clones secreting a neutralizing antibody. Of these clones, three were isolated from donor CL26, one from donor CL114, and eleven from donor CL115. The number of clones isolated from each donor did not correlate with the plasma titer of neutralizing antibody, though this was not surprising given the small sample size. Clones producing antibodies that recognized H5 HA by ELISA, but did not neutralize live virus, were also identified from each donor (unpublished data). Clones FLA3.14 and FLA5.10, isolated from donor CL26, were the first obtained and were studied more extensively. Subsequently, clones FLD20.19 and FLD21.140, isolated from donor CL115, became available and were studied in parallel with FLA3.14 and FLA5.10. Clones FLA3.14, FLA5.10, FLD20.19, and FLD21.140 secreted IgG1,κ antibodies with neutralizing activity against the autologous virus A/Vietnam/CL26/2004 and other more recent Clade I viruses circulating in Viet Nam during 2005 and 2006 (Table 1). Significantly, more distant HPAI H5N1 strains, including the Clade II H5N1 virus A/Indonesia/5/2005, were neutralized by FLA3.14 and FLD20.19 (Table 2). In contrast, none of these clones neutralized an H3N2 influenza virus, A/California/7/2004 (Table 2). IgG1,κ mAbs of irrelevant specificity (diphtheria toxin and anthrax protective antigen) were used as negative controls and did not neutralize any influenza virus (Tables 1 and 2). Thus, the mAbs selected for further study demonstrated broad in vitro neutralizing activity against H5N1 viruses isolated from 1997 to 2005, albeit with some variation in potency.

**Table 1:**
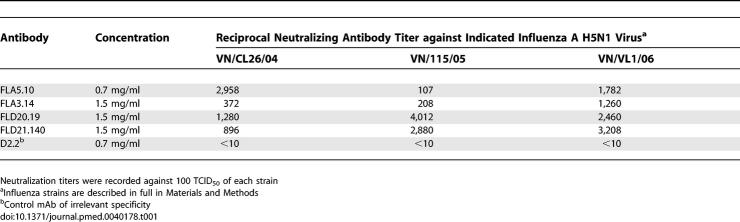
Neutralizing Titers of mAbs against Influenza A H5N1 Influenza Viruses Isolated from Viet Nam

**Table 2:**
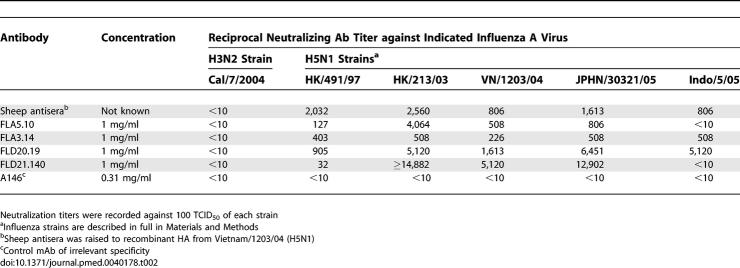
Neutralizing Titers of mAbs against Influenza A H3N2 and a Range of H5N1 Influenza Reference Viruses

**Figure 1:**
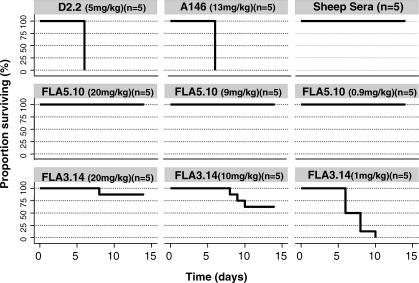
Passive Immunization and Survival after Challenge with A/Vietnam/1203/04 (H5N1) BALB/c mice (*n* = 5 per group) were passively immunized by i.p. injection of graded doses of anti-H5N1 mAbs FLA3.14 or FLA5.10, the control mAbs D2.2 or A146, or hyperimmune sheep antisera specific for the H5N1 HA protein. Mice were challenged i.n. with 50 μl of A/Vietnam/1203/04 (10^5^ TCID_50_/mouse) 24 h later. The data show the Kaplan-Meier survival curves for the 2 wk period of observation. Mice that received either sheep anti-H5 antisera or FLA5.10 were completely protected from lethal infection (sheep antisera or FLA5.10 versus D2.2, *p* = 0.001, log rank test). FLA3.14 afforded significant protection at 20 mg/kg and 10 mg/kg (FLA3.14 versus D2.2, *p* = 0.001, log rank test). The lowest dose of FLA3.14 (1 mg/kg) delayed time to death (FLA3.14 versus D2.2, *p* = 0.02, log rank test), but did not prevent fatal infection.

BALB/c mice are highly susceptible to infection with the HPAI H5N1 viruses isolated in Asia in 1997 and since 2003. Following i.n. administration, these H5N1 viruses replicate to high titer in the lungs of the mice and some isolates disseminate to extrapulmonary sites and are lethal for mice [22,23]. To explore the efficacy of mAbs FLA3.14 and FLA5.10 for pre-exposure prophylaxis, BALB/c mice were passively immunized by i.p. administration of graded doses of mAbs and then challenged i.n. with A/Vietnam/1203/04 (H5N1) 24 h later. All preparations of mAb FLA5.10 conferred 100% protection from lethality by A/Vietnam/1203/04 (*p* = 0.001) (Figure 1). mAb FLA3.14 also conferred some protection from lethal A/Vietnam/1203/04 (H5N1) infection, but with lower efficacy and in a dose-dependent manner (Figure 1). Mice receiving the highest dose of FLA3.14 were afforded almost complete protection (*p* = 0.001), whilst mice receiving the lowest dose of FLA3.14 were as susceptible as mice that received a human mAb of irrelevant specificity, though time to death was delayed (*p* = 0.02). Mice that received hyperimmune anti-H5 polyclonal sheep antiserum were afforded complete protection. These data, demonstrating the relatively greater in vivo activity of FLA5.10 over FLA3.14 against A/Vietnam/1203/04, are consistent with the in vitro neutralization titers presented in Table 1.

**Figure 2:**
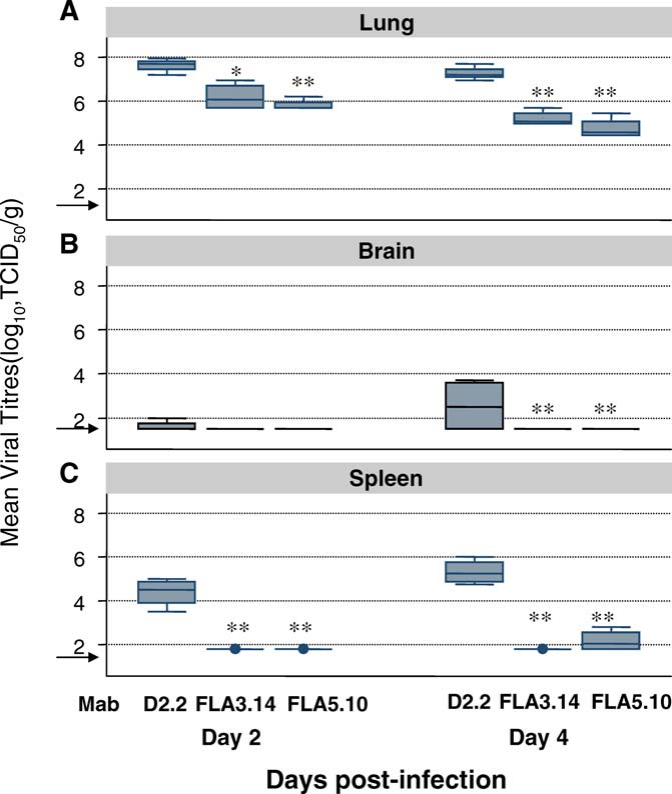
Pulmonary Virus Titers and Extrapulmonary Dissemination of A/Vienam/1203/04 (H5N1) Following Passive Immunization The data depict the mean (± interquartile range) viral titer in (A) lungs, (B) brain, and (C) spleen of groups of five BALB/c mice passively immunized by i.p. injection of mAbs FLA3.14, FLA5.10, or the control mAb D2.2, then challenged i.n. with 50 μl of A/Vietnam/1203/04 (10^5^ TCID_50_/mouse) 24 h later. On days 2 and 4 there was significantly less virus recovered in splenic and pulmonary tissue of mice that had received either FLA3.14 or FLA5.10 than mice that had received the control mAb, D2.2. (* *p* < 0.01 versus D2.2; ** *p* < 0.001 versus D2.2). The lower limit of detection (1.5 log_10_ TCID_50_/g) is shown by the arrow.

**Figure 3:**
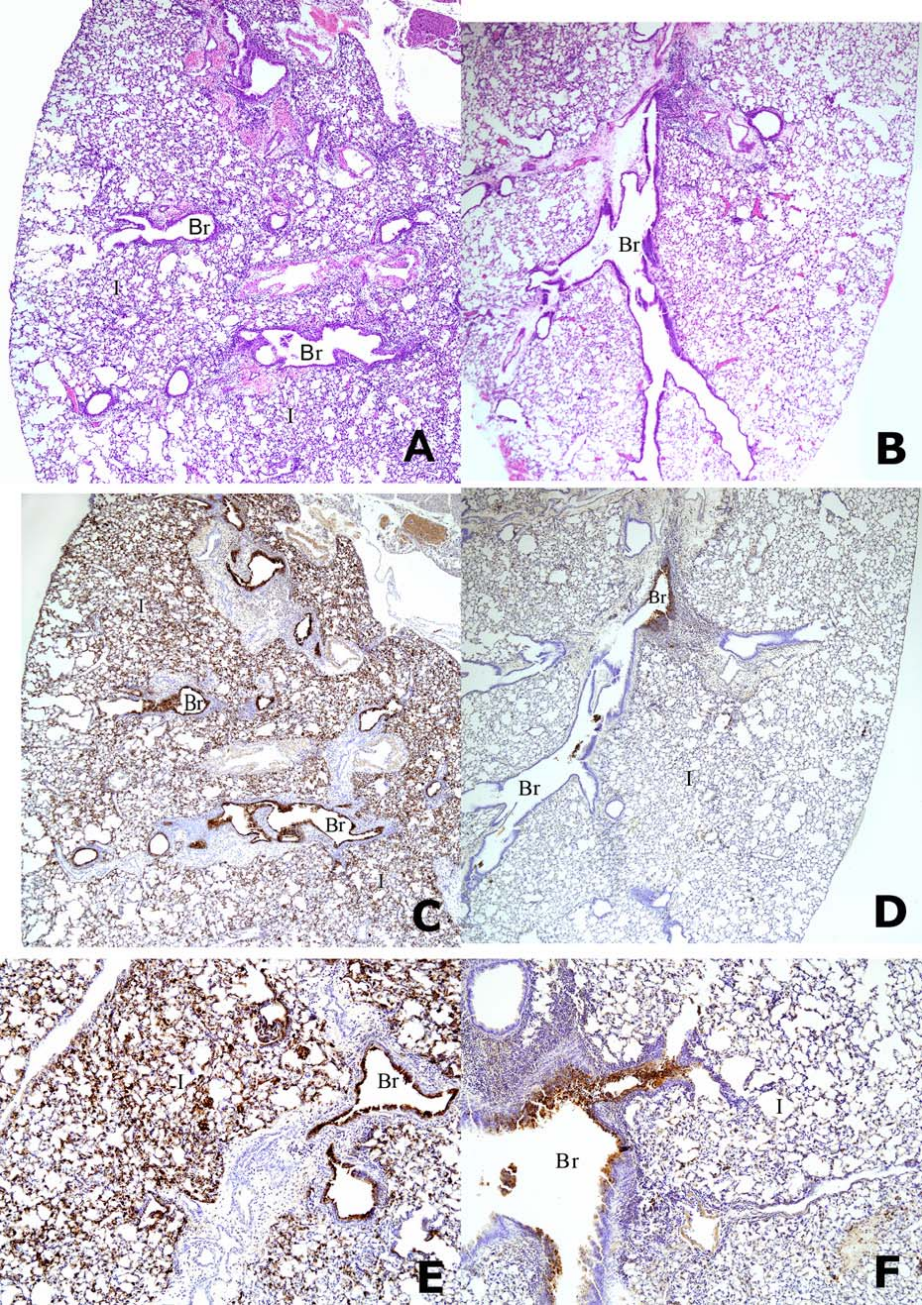
Histopathology in Pulmonary Tissue of Passively Immunized and Challenged Mice (A) Hematoxylin and eosin-stained lung sections (40×) revealed diffuse interstitial pneumonia (I) and bronchial and bronchiolar (Br) involvement in a mouse infected with influenza A/VN/1203/04 (H5N1) after i.p. injection of the control mAb D2.2. (B) Mouse given mAb FLA5.10 prior to infection with influenza A/VN/1203/04 (H5N1), showing much less lung involvement than in (A). (C) Immunohistochemistry (40×) revealed H5 antigen in bronchi, bronchioles (Br), and interstitial lesions (I) in a mouse given influenza A/VN/1203/04 (H5N1) following i.p. injection of the control mAb D2.2. (D) H5 antigen in bronchus (Br) and not in bronchioles or interstitial areas (I) in a mouse given mAb FLA5.10 prior to influenza challenge. (E) High magnification (100×) of (C) showing abundant H5 antigen in interstitial alveolar lesions (I) and bronchiolar epithelium (Br). (F) High magnification of (D) (100×) showing H5 antigen only focally in a bronchus (Br) and not in the interstitial alveolar areas (I).

To better understand the mechanism by which mAbs FLA3.14 and FLA5.10 conferred protection from lethality, the kinetics of viral infection in passively immunized mice was defined. Mice that were passively immunized with FLA3.14, FLA5.10, or a human mAb of irrelevant specificity (D2.2) were challenged with A/Vietnam/1203/04 (H5N1) 24 h later, and the level of virus replication in different organs was determined 2 and 4 d later. Mice that received the control mAb, D2.2, had high titers of virus in the lungs (Figure 2A), with evidence of extrapulmonary dissemination indicated by viral replication in the brain (Figure 2B) and spleen (Figure 2C). In contrast, mice passively immunized with FLA3.14 or FLA5.10 had significantly (10- to 100-fold) lower titers of virus in the lungs (Figure 2A) (*p* = 0.01, FLA3.14 versus D2.2; *p* = 0.001, FLA5.10 versus D2.2), undetectable viral burdens in the brain (Figure 2B) and a low titer of virus detected in the spleens of mice that received FLA5.10 (Figure 2C). Alongside the reduction in lung viral titers, mice that received prophylaxis with FLA5.10 had less dramatic pathological changes in the pulmonary airways and parenchymal tissue (Figure 3). Thus, the percentage of abnormal bronchioles with necrosis and viral antigen in lung sections from mice (*n* = 2 per group) that received FLA5.10 prophylaxis was less (13%) than in control mice (80%). Similarly, there were fewer inflammatory interstitial lesions in which H5 antigen was detected by immunohistochemical staining in the lung sections of mice given FLA5.10 relative to the control antibody, D2.2 (1 versus >10) (Figure 3). To a slightly lesser extent, FLA3.14 prophylaxis also limited bronchiolitis (31% versus 80%) and H5-associated interstitial pathology (2 versus >10) when compared with control mice (*n* = 2). These data suggest that prophylaxis with FLA3.14 or FLA5.10 probably confers protection from lethal challenge through a combination of limiting viral replication in the lung, attenuating virus-induced lung pathology, and blocking extrapulmonary dissemination of virus to distant organs.

**Figure 4:**
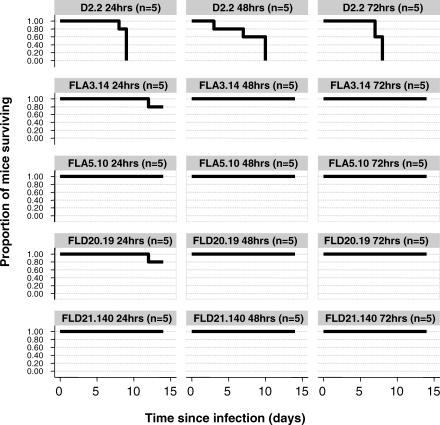
mAb Therapy and Survival in Mice with Established A/Vietnam/1203/04 (H5N1) Infection The data show the Kaplan-Meier survival curves for groups of BALB/c mice (*n* = 5 per group) that were infected i.n. with 5 LD_50_ of A/Vietnam/1203/04, then 24, 48, or 72 h later treated by i.p. injection with the control mAb D2.2, or the anti-H5N1 mAbs FLA3.14, FLA5.10, FLD20.19, or FLD21.140, each at 50 mg/kg body weight. Postinfection therapy at 24, 48, or 72 h with mAbs FLA3.14, FLA5.10, FLD20.19, or FLD21.140 was associated with significant protection from lethal infection at all time points (FLA3.14, FLA5.10, FLD20.19, or FLD21.140 versus D2.2, *p* = 0.003, log rank test).

**Figure 5:**
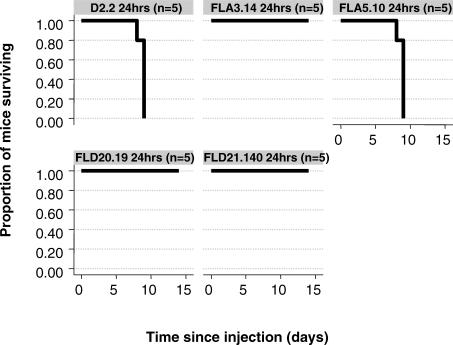
mAb Therapy and Survival in Mice with Established A/Indonesia/5/2005 (H5N1) Infection The data show the Kaplan-Meier survival curves for groups of BALB/c mice (*n* = 5 per group) that were infected i.n. with 5 LD_50_ of A/Indonesia/5/2005 (H5N1) then 24 h later treated by i.p. injection with the control mAb D2.2, or the anti-H5N1 mAbs FLA3.14, FLA5.10, FLD20.19, or FLD21.140, each at 50 mg/kg body weight. Postinfection therapy at 24 h with mAbs FLA3.14, FLD20.19, or FLD21.140, but not FLA5.10 or D2.2, was associated with absolute protection from lethal infection (FLA3.14, FLD21.140 or FLD20.19 versus D2.2, *p* = 0.003, log rank test).

Attenuation of established infection represents a clinically relevant endpoint for antiviral therapy against H5N1 infection. To this end, the efficacy of treatment with FLA3.14, FLA5.10, FLD20.19, and FLD21.140 was determined in BALB/c mice i.n. infected 24, 48, or 72 h previously with 5 LD_50_ of A/Vietnam/1203/04 (H5N1). FLA3.14, FLA5.10, FLD20.19, and FLD21.140 provided robust protection from lethality in A/Vietnam/1203/04 (H5N1) infected mice at all time points, whilst an irrelevant control mAb, D2.2, gave no protection (*p* = 0.003) (Figure 4). These promising therapeutic results against a Clade I virus from Viet Nam led us to examine the therapeutic efficacy of mAbs FLA3.14, FLA5.10, FLD20.19, and FLD21.140 against A/Indonesia/5/2005, an antigenically divergent H5N1 virus from Clade II. The efficacy of treatment with FLA3.14, FLA5.10, FLD20.19, and FLD21.140 was determined in BALB/c mice infected i.n. 24 h previously with 5 LD_50_ of A/Indonesia/5/2005. Consistent with the in vitro neutralization data (Table 2), mice treated with FLA3.14 and FLD20.19, but not FLA5.10 or the control mAb D2,2, were significantly protected from A/Indonesia/5/2005 lethal infection (*p* = 0.003) (Figure 5). Although FLD21.140 did not demonstrate neutralizing activity in vitro against A/Indonesia/5/2005 (Table 2), treatment with this mAb significantly protected the mice (*p* = 0.003) from A/Indonesia/5/2005 lethal infection. This observation of neutralization in vivo suggests that a factor found in vivo enhances the neutralizing activity of this mAb, accounting for its efficacy in vivo in preventing mortality associated with infection. These data provide proof of concept that mAb therapy for at least 72 h postinfection in the mouse model can markedly improve survival from highly virulent H5N1 infection. Importantly, these data also imply it is possible to obtain significant cross-protection against a Clade II H5N1 virus using a mAb elicited by a Clade I virus.

## Discussion

The risk of a devastating human influenza pandemic caused by an H5N1 influenza strain remains difficult to quantify. What is clear is that zoonotic infections with HPAI H5N1 viruses continue to occur in Southeast Asia with a mortality in 2006 of 67% and for which there are few specific interventions [20,24]. Here we report on the generation of four fully human mAbs with a spectrum of neutralizing activity against multiple strains of HPAI H5N1 viruses in vitro and in vivo. These mAbs could have potential in the adjunctive treatment of human pandemic or zoonotic H5N1 cases.

Prophylaxis with mAb FLA5.10, and to a lesser extent FLA3.14, conferred significant immunity to mice infected with A/Vietnam/1203/04. Passive immunity was associated with significantly reduced viral burdens in the lung and negligible dissemination of virus to the brain or spleen. Viral dissemination might be an important aspect in the pathogenesis of H5N1 viruses in both mice and humans. In Vietnamese patients with H5N1 infection, fatal outcomes were strongly associated with the presence of viral genetic material, or viable virus, at extrapulmonary sites [3]. Conversely, there was no evidence of extrapulmonary virus dissemination in patients who survived [3]. In addition to evidence of viral dissemination, patients with fatal H5N1 infections had high viral loads in the respiratory tract, hypercytokinemia, multiple organ dysfunction, and acute respiratory distress syndrome [3]. Our proposed model of H5N1 pathogenesis [3] argues that early diagnosis and antiviral interventions that limit the ensuing inflammatory cascade should be central to treatment.

Although not a new strategy, antibody-based therapy for severe influenza caused by H5N1 viruses represents a plausible intervention. Multiple reports of physicians using human blood products from recovering influenza patients appeared during the 1918 Spanish H1N1 influenza pandemic. A recent review of these studies suggested this treatment was associated with a halving in mortality (37% to 16%), and that early treatment was associated with greater benefits [16]. The assumption underlying these observations is that neutralizing antiviral antibodies in the plasma preparations modulated the course of viral infection and thereby prevented the development of acute respiratory distress syndrome and other complications [16]. These same tenets form the rationale for therapy using the mAbs generated in this study, with the potential of a scaleable therapeutic product free of adventitious agents. The strengths of the approach for human mAb generation described here are: (1) it uses the human immune response rather than that of animal surrogates—the antibodies selected will be those that have been generated in response to the natural infectious pathogen and have protected the individual, (2) it is fast, (3) screening can be performed using functional assays, i.e., neutralization, (4) it allows screening of a large repertoire of antibodies to select those with the most favorable profile (potency and breadth of reactivity), and (5) since the antibodies are of human origin the risks of self-reactivity against self-antigens is minimized when compared with antibodies generated in mice or through phage display.

The mAbs produced in this study were derived from immortalized memory B cells of donors who had recovered from H5N1 infection. Overall, compared with the yield of neutralizing B cell clones we previously derived from patients who had recovered from SARS coronavirus infection [19], donor-derived B cell clones that neutralized H5N1 influenza were relatively scarce. These observations might reflect the weak immunogenicity of the H5 HA, as suggested in trials of inactivated H5N1 vaccines in human volunteers [25].

Two of the four mAbs characterised in this study had cross-reactive antiviral activity in vitro and in vivo against Clade I and Clade II H5N1 viruses. This is significant, as it suggests the presence of conserved neutralizing epitopes on representatives of these two clades. One mAb (FLD21.140) was effective in neutralizing a Clade II virus in vivo but not in vitro, suggesting the neutralizing activity of this mAb is dependent upon a factor found in vivo, such as complement. Similar findings have been previously reported by Gerhard and colleagues who identified a mouse mAb against an influenza A H1 HA that had neutralizing activity in vivo but not in vitro [26,27]. The in vitro neutralizing activity of this mAb was enhanced by the C1q component of the complement system plus another undefined serum factor [26,27].

It was possible to employ the human mAbs generated in this study as potent therapeutic agents for at least 72 h after A/Vietnam/1203/04 infection. This is important as most zoonotic cases of human H5N1 infection do not present to health care facilities until at least several days after illness onset [3]. Potentially, a cocktail of these cross-reactive mAbs could represent an adjunctive treatment option against H5N1 infection. The dose of mAb required for effective anti-influenza H5N1 activity in a patient is uncertain, though we note that the only mAb licensed for use against a viral agent (respiratory syncytial virus) is used at 15 mg/kg of body weight.

The ongoing process of antigenic variation in antibody binding sites, called antigenic drift, in influenza viruses represents a challenge to vaccine design and also to therapy using mAbs. Although the molecular targets of the neutralizing mAbs in this study have not been determined, they are presumed to reside around the highly variable receptor binding region of HA1 [28–30] and that differences in potency are related to epitope specificity and overall avidity. To date, we have not identified mAb epitope escape mutants of H5N1. These issues, and the mechanism of virus neutralization, are subjects of ongoing virological and crystallographic studies.

HPAI H5N1 viruses continue to circulate and evolve in bird populations. It is not certain that a pandemic virus originating from an HPAI H5N1 virus will resemble the H5N1 viruses studied here, or that the mAbs generated here will have neutralizing activity against a pandemic virus. Nevertheless, we are encouraged by the broad neutralizing activity of these antibodies in vitro, and the moderate doses required in vivo to confer protection. Ultimately, we hope that these mAbs, and others like them, could constitute a cocktail of cross-reactive, neutralizing antibodies that could be employed as adjunctive treatment of H5N1 influenza.

## Supporting Information

### Accession Numbers

The GenBank (http://www.ncbi.nlm.nih.gov/) HA sequences of the H5N1 viruses discussed in this paper are A/Vietnam/CL115/2005 (DQ497727), A/Vietnam/CL26/2004 (DQ497723), and A/Vietnam/CL36/2005 (DQ497724).

## Abbreviations

EBV: Epstein-Barr virus
HA: hemagglutinin
HPAI: highly pathogenic avian influenza H5N1 viruses
Ig: immunoglobulin
i.n.: intranasal(ly)
i.p.: intraperitoneal(ly)
mAb: monoclonal antibody
PBMC: peripheral blood mononuclear cell
TCID_50_: 50% tissue culture infectious dose(s)
wt: wild type

## References

[pmed-0040178-b001] World Health Organization Global Influenza Program Surveillance Network (2005). Evolution of H5N1 avian influenza viruses in Asia. Emerg Infect Dis.

[pmed-0040178-b002] No Authors Listed (2006). Epidemiology of WHO-confirmed human cases of avian influenza A(H5N1) infection. Wkly Epidemiol Rec.

[pmed-0040178-b003] de Jong MD, Simmons CP, Thanh TT, Hien VM, Smith GJ (2006). Fatal outcome of human influenza A (H5N1) is associated with high viral load and hypercytokinemia. Nat Med.

[pmed-0040178-b004] Li KS, Guan Y, Wang J, Smith GJ, Xu KM (2004). Genesis of a highly pathogenic and potentially pandemic H5N1 influenza virus in eastern Asia. Nature.

[pmed-0040178-b005] Puthavathana P, Auewarakul P, Charoenying PC, Sangsiriwut K, Pooruk P (2005). Molecular characterization of the complete genome of human influenza H5N1 virus isolates from Thailand. J Gen Virol.

[pmed-0040178-b006] Le QM, Kiso M, Someya K, Sakai YT, Nguyen TH (2005). Avian flu: Isolation of drug-resistant H5N1 virus. Nature.

[pmed-0040178-b007] de Jong MD, Tran TT, Truong HK, Vo MH, Smith GJ (2005). Oseltamivir resistance during treatment of influenza A (H5N1) infection. N Engl J Med.

[pmed-0040178-b008] Sawyer LA (2000). Antibodies for the prevention and treatment of viral diseases. Antiviral Res.

[pmed-0040178-b009] Smirnov YA, Lipatov AS, Gitelman AK, Claas EC, Osterhaus AD (2000). Prevention and treatment of bronchopneumonia in mice caused by mouse-adapted variant of avian H5N2 influenza A virus using monoclonal antibody against conserved epitope in the HA stem region. Arch Virol.

[pmed-0040178-b010] Renegar KB, Small PA, Boykins LG, Wright PF (2004). Role of IgA versus IgG in the control of influenza viral infection in the murine respiratory tract. J Immunol.

[pmed-0040178-b011] Palladino G, Mozdzanowska K, Washko G, Gerhard W (1995). Virus-neutralizing antibodies of immunoglobulin G (IgG) but not of IgM or IgA isotypes can cure influenza virus pneumonia in SCID mice. J Virol.

[pmed-0040178-b012] Puck JM, Glezen WP, Frank AL, Six HR (1980). Protection of infants from infection with influenza A virus by transplacentally acquired antibody. J Infect Dis.

[pmed-0040178-b013] Sweet C, Bird RA, Jakeman K, Coates DM, Smith H (1987). Production of passive immunity in neonatal ferrets following maternal vaccination with killed influenza A virus vaccines. Immunology.

[pmed-0040178-b014] Sweet C, Jakeman KJ, Smith H (1987). Role of milk-derived IgG in passive maternal protection of neonatal ferrets against influenza. J Gen Virol.

[pmed-0040178-b015] Reuman PD, Paganini CM, Ayoub EM, Small PA (1983). Maternal-infant transfer of influenza-specific immunity in the mouse. J Immunol.

[pmed-0040178-b016] Luke TC, Kilbane EM, Jackson JL, Hoffman SL (2006). Meta-analysis: Convalescent blood products for Spanish influenza pneumonia: A future H5N1 treatment?. Ann Intern Med.

[pmed-0040178-b017] Lu J, Guo Z, Pan X, Wang G, Zhang D (2006). Passive immunotherapy for influenza A H5N1 virus infection with equine hyperimmune globulin F(ab')2 in mice. Respir Res.

[pmed-0040178-b018] Hanson BJ, Boon AC, Lim AP, Webb A, Ooi EE (2006). Passive immunoprophylaxis and therapy with humanized monoclonal antibody specific for influenza A H5 hemagglutinin in mice. Respir Res.

[pmed-0040178-b019] Traggiai E, Becker S, Subbarao K, Kolesnikova L, Uematsu Y (2004). An efficient method to make human monoclonal antibodies from memory B cells: Potent neutralization of SARS coronavirus. Nat Med.

[pmed-0040178-b020] Tran TH, Nguyen TL, Nguyen TD, Luong TS, Pham PM (2004). Avian influenza A (H5N1) in 10 patients in Vietnam. N Engl J Med.

[pmed-0040178-b021] Rowe T, Abernathy RA, Hu-Primmer J, Thompson WW, Lu X (1999). Detection of antibody to avian influenza A (H5N1) virus in human serum by using a combination of serologic assays. J Clin Microbiol.

[pmed-0040178-b022] Lu X, Tumpey TM, Morken T, Zaki SR, Cox NJ (1999). A mouse model for the evaluation of pathogenesis and immunity to influenza A (H5N1) viruses isolated from humans. J Virol.

[pmed-0040178-b023] Gao P, Watanabe S, Ito T, Goto H, Wells K (1999). Biological heterogeneity, including systemic replication in mice, of H5N1 influenza A virus isolates from humans in Hong Kong. J Virol.

[pmed-0040178-b024] Beigel JH, Farrar J, Han AM, Hayden FG, Hyer R (2005). Avian influenza A (H5N1) infection in humans. N Engl J Med.

[pmed-0040178-b025] Treanor JJ, Campbell JD, Zangwill KM, Rowe T, Wolff M (2006). Safety and immunogenicity of an inactivated subvirion influenza A (H5N1) vaccine. N Engl J Med.

[pmed-0040178-b026] Feng JQ, Mozdzanowska K, Gerhard W (2002). Complement component C1q enhances the biological activity of influenza virus hemagglutinin-specific antibodies depending on their fine antigen specificity and heavy-chain isotype. J Virol.

[pmed-0040178-b027] Mozdzanowska K, Feng J, Eid M, Zharikova D, Gerhard W (2006). Enhancement of neutralizing activity of influenza virus-specific antibodies by serum components. Virology.

[pmed-0040178-b028] Wilson IA, Skehel JJ, Wiley DC (1981). Structure of the haemagglutinin membrane glycoprotein of influenza virus at 3 A resolution. Nature.

[pmed-0040178-b029] Wiley DC, Wilson IA, Skehel JJ (1981). Structural identification of the antibody-binding sites of Hong Kong influenza haemagglutinin and their involvement in antigenic variation. Nature.

[pmed-0040178-b030] Wilson IA, Cox NJ (1990). Structural basis of immune recognition of influenza virus hemagglutinin. Annu Rev Immunol.

